# Ureidothiophene inhibits interaction of bacterial RNA polymerase with –10 promotor element

**DOI:** 10.1093/nar/gkaa591

**Published:** 2020-07-11

**Authors:** John Harbottle, Nikolay Zenkin

**Affiliations:** Centre for Bacterial Cell Biology, Biosciences Institute, Faculty of Medical Sciences, Newcastle University, Baddiley-Clark Building, Richardson Road, Newcastle Upon Tyne NE2 4AX, UK; Centre for Bacterial Cell Biology, Biosciences Institute, Faculty of Medical Sciences, Newcastle University, Baddiley-Clark Building, Richardson Road, Newcastle Upon Tyne NE2 4AX, UK

## Abstract

Bacterial RNA polymerase is a potent target for antibiotics, which utilize a plethora of different modes of action, some of which are still not fully understood. Ureidothiophene (Urd) was found in a screen of a library of chemical compounds for ability to inhibit bacterial transcription. The mechanism of Urd action is not known. Here, we show that Urd inhibits transcription at the early stage of closed complex formation by blocking interaction of RNA polymerase with the promoter –10 element, while not affecting interactions with –35 element or steps of transcription after promoter closed complex formation. We show that mutation in the region 1.2 of initiation factor σ decreases sensitivity to Urd. The results suggest that Urd may directly target σ region 1.2, which allosterically controls the recognition of –10 element by σ region 2. Alternatively, Urd may block conformational changes of the holoenzyme required for engagement with –10 promoter element, although by a mechanism distinct from that of antibiotic fidaxomycin (lipiarmycin). The results suggest a new mode of transcription inhibition involving the regulatory domain of σ subunit, and potentially pinpoint a novel target for development of new antibacterials.

## INTRODUCTION

Bacterial multi-subunit DNA-dependent RNA polymerase (RNAP) is a proven target for a number of antibacterial compounds. About a half of these, including the only two compounds used clinically (fidaxomicin and compounds of the rifamycin class), specifically target transcription initiation. During initiation of transcription, the RNAP holoenzyme (core RNAP joined by initiation factor σ) binds to promoter DNA to form a ‘closed promoter complex’ (RPc) in which the RNAP is bound to double stranded promoter DNA. For RPc to form, conserved substructures of σ in the context of holoenzyme must interact with promoter DNA. σ subunit regions 2 (σR2) and 4.2 (σR4) recognize the core –10 and –35 promoter elements, respectively (1). Alternatively, σR2 and σR3.0 recognize the extended –10 motif (2). σR2 nucleates promoter melting by intercalating into the –10 promoter element at the –12 position (3). Conserved non-template DNA bases at the −11 and −7 positions are flipped out of the DNA duplex into protein pockets on σR2, stimulating interaction of downstream double-stranded DNA with RNAP, propagation of the transcription bubble, and loading of single-stranded template DNA into the active center of RNAP, i.e. formation of ‘open promoter complex’ RPo (1,4). The highly conserved σ subunit region 1.2 (σR1.2, residues ∼96–127) plays a key role in RPo formation. Holoenzymes lacking σR1.2 are unable to recognize single stranded –10 promoter sequence DNA (5,6), while certain substitutions in σR1.2 make the holoenzyme incapable of forming stable open complexes and, thus, highly defective in transcription initiation (7). It is hypothesized that σR1.2 directly or allosterically stabilizes the optimal conformation of σR2 required for –10 promoter element recognition (4,6). Furthermore, σR1.2 interacts with non-template promoter DNA downstream of the –10 element within the ‘discriminator’ region (4,8,9), contributing to stability of RPo. At the beginning of RNA synthesis, σR1.2 facilitates melting of double-stranded DNA thus allowing translocation of RNAP (10).

Specific inhibitors of initiation of transcription include Fidaxomicin (Fdx), Ripostatin (Rip), Myxopyronin (Myx), Corallopyronin (Cor), GE23077, rifamycins (Rifs), and ureidothiophene (Urd). Fdx blocks RPo (and probably correct RPc) formation by locking the RNAP clamp in an open conformation by binding to the switch region 2 of β′ subunit of RNAP (β′ switch-2), the molecular hinge that facilitates clamp movement (11). Fdx does not affect the binding of upstream promoter elements whilst destabilizing binding of downstream promoter DNA (12). Rip, Cor and Myx also binds to the β′ switch-2 but inhibits isomerization to RPo at a later stage than Fdx by trapping a promoter complex with a partially melted transcription bubble that fails to propagate to the transcription start site (13,14). All the inhibitors binding at the β′ switch-2, affect loading of the single-stranded template DNA in the RNAP active center during RPo formation. GE23077 binds to β-subunit and competes with the very first (+1) initiating nucleotide in the i-site of the RNAP active center (15). Rifamycins bind to the β subunit and inhibit first phosphodiester bond formation and/or translocation of the nascent di- or trinucleotide-long nascent RNA (reviewed in (16)). Urd was proposed to inhibit bacterial transcription at the initiation step (17), though the mechanism of this action is not known.

**Figure 1.**
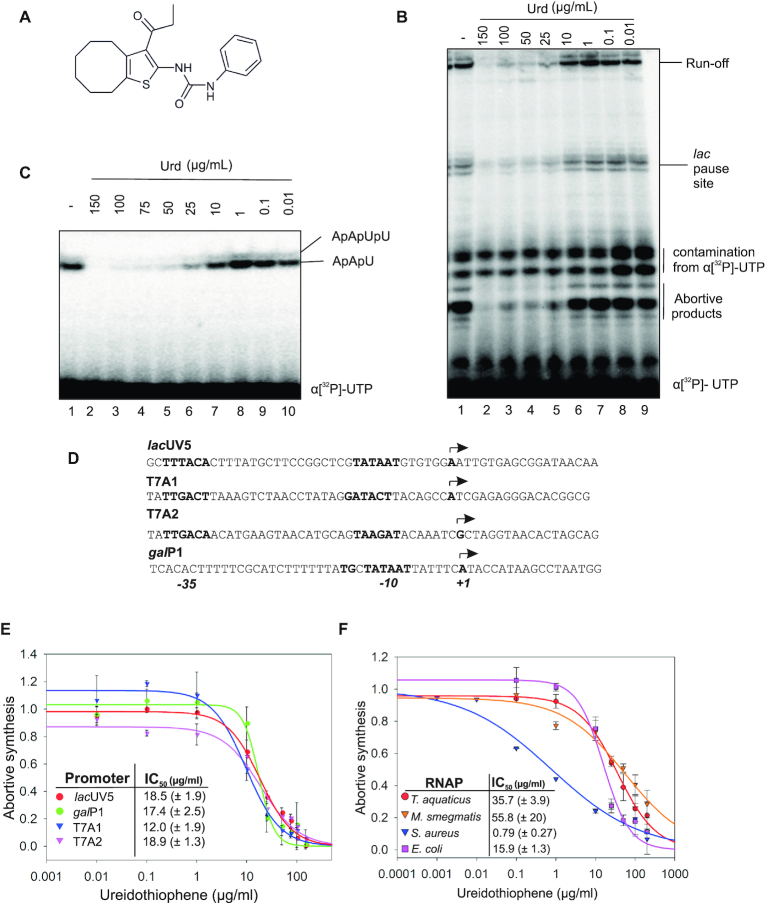
Ureidothiophene (Urd) inhibits bacterial RNA polymerases. (**A**) Structural formula of Urd. (**B**) Urd inhibition of *in vitro* transcription by *E. coli* RNAP on a linear DNA template containing the *lac*UV5 promoter. (**C**) Urd inhibition of abortive synthesis by *E. coli* RNAP on *lac*UV5 promoter. (**D**) Sequence of promoters used in panel E. Promoter elements and TSS are indicated. (**E**) Urd inhibition of *in vitro* transcription by *E. coli* RNAP on promoters shown in panel D. Error bars are ± SD. (**F**) Urd inhibition of *in vitro* transcription by bacterial RNAP holoenzymes from different bacteria on T7A1 promoter. Error bars are ± SD.

Urd is a synthetic RNAP inhibitor (Figure 1A) that was discovered in a high-throughput screen of chemical compounds for inhibitory activity against *Staphylococcus aureus* RNAP holoenzyme *in vitro*. Urd was shown to be highly active against *S. aureus* RNAP *in vitro*, with an IC_50_ of ∼1 μM (18). Furthermore, Urd possessed a narrow spectrum of activity against *S. aureus* ATCC 13709 and *S. epidermidis* with a ∼MIC of 1 and 0.25 μg/ml, respectively (18). An isopropyl derivative of Urd was shown to inhibit RNA and protein synthesis, but not DNA synthesis by *S. aureus* RN4220 strain (18). Additionally, the compound retained activity against Rif resistant strains of *S. aureus* suggesting the binding site of Urd is different to that of Rif (18).

Here we show that Urd inhibits formation of RPc by blocking interactions of holoenzyme with DNA downstream –35 promoter element and potentially by targeting σR1.2, the regulator of the recognition of the –10 promoter element by σR2.

## MATERIALS AND METHODS

### Antibiotics and DNA templates

All chemicals and reagents were purchased from Sigma unless otherwise stated. Ureidothiophene was purchased from ChemBridge™. All promoter DNA fragments were produced by PCR using Phusion DNA polymerase from their respective primers (IDT) and purified by agarose gel electrophoresis (Qiagen). lacUV5-promoter fragment was produced by PCR with the primers 5′-CTCACTCATTAGGCACCCCAGGC-3′ and 5′-CCAGGCGGTGAAGGGCAATCAGC-3′ from template CTCACTCATTAGGCACCCCAGGCTTTACACTTTATGCTTCCGGCTCGTATAATGTGTGGAATTGTGAGCGGATAACAATTTCACACAGGAAACAGCTGATTGCCCTTCACCGCC. *T7A1*-promoter fragment was produced by PCR with the primers 5′-GGTCGACTCTAGAGGATCGCT-3′ and 5′-CGACGTTGTAAAACGACGGCCAGTG from template GGTCGACTCTAGAGGATCGCTATAACAGGCCTGCTGGTAATCGCAGGCCTTTTTATTTGGATCCAGATCCCGAAAATTTATCAAAAAGAGTATTGACTTAAAGTCTAACCTATAGGATACTTACAGCCATCGAGAGGGACACGGCGAATAGCCATCCCAATCGACACCGGGGTCCGGGATCTGGATCTGGATCGCTAATAACAGGCCTGCTGGTAATCGCAGGCCTTTTTATTTGGATCCCCGGGTACCGAGCTCGAATTCACTGGCCGTCGTTTTACAACGTCG. *T7A2*-promoter fragment was produced by PCR with the primers 5′-TCGACACCGGGGGAATTCGG and 5′-CGCTTAAGTCACCTAGAAGGC from template TCGACACCGGGGGAATTCGGATAAGTAGACAGCCTGATAAGTCGCACGAAAAACAGGTATTGACAACATGAAGTAACATGCAGTAAGATACAAATCGCTAGGTAACACTAGCAGCGTCAACCGGGCGCGCAGTGCCTTCTAGGTGACTTAAGCG. *galP1*-promoter fragment was produced by PCR with the primers 5′-GGCTAAATTCTTGTGTAAACGATTCCA-3′ and 5′-CTCATAATTCGCTCCATTAGGCTTATG-3′ from template GGCTAAATTCTGTGTAAACGATTCCACTAATTTATTCCATGTCACACTTTTCGCATCTTTTTTATGCTATAATTATTTCATACCATAAGCCTAATGGAGCGAATTATGAG. M13 single-stranded promoter was from IDT.

### Protein expression and purification

Core *E. coli* RNAP subunits were expressed in T7 express cells (New England Biolabs) transformed with pGEMABC (encoding rpoA, rpoB and rpoC) and pACYCDuet-1_Ec_rpoZ (encoding rpoZ) (19). Expression was induced by addition of 0.4 mM final IPTG to exponentially growing cells and incubated on an orbital shaker (150 rpm) at room temperature overnight. Cells were then harvested by centrifugation and resuspended in grinding buffer (50 mM Tris–HCl (pH 7.9), 10% glycerol, 200 mM NaCl and protease inhibitor mixture). Cells were then lysed by sonication and debris cleared by centrifugation. RNAP was precipitated from the lysate by addition of polyethyleneimine solution to a final concentration of 0.6% and the pellet recovered by centrifugation. RNAP was eluted from the pellet by suspension in TGED buffer (10 mM Tris–HCl (pH 7.0), 10% glycerol, 0.1 mM EDTA and 0.1 mM DTT) + 1 M NaCl and then precipitated by ammonium sulfate to a final concentration of 60% saturation. The pellet was resuspended in TGED buffer + 50 mM NaCl. Core RNAP was purified by HiTrap Heparin affinity chromatography followed by ion-exchange chromatography on Resource Q column (GE Healthcare).

Cellular RNAP core from *S. aureus* SH1000 was purified in the same way except the cells were grown in Brain-Heart Infusion liquid medium.

Cellular His-tagged *M. smegmatis* RNAP σ^A^ holoenzyme was purified as described previously (20). *M. smegmatis* SM07 cells were grown in 7H9 supplemented with 0.25% glycerol, 1% glucose, 0.04% tyloxapol and 50 μg/ml carbenicillin to OD_600_ = 0.8. Cells were pelleted and disrupted as above. Nickel-affinity chromatography (GE Healthcare) was followed by ion exchange chromatography on Resource Q column (GE Healthcare), where σ^A^ holoenzyme was collected. Identity of σ^A^ was confirmed by mass spectrometry.

Recombinant *T. aquaticus* was purified as described previously (21). SDS-gels of above purified RNAPs are shown in Supplementary Figure S1.

Cellular *S. epidermidis* wild-type and E105Q σ^A^ RNAP holoenzymes were purified from respective strains of wild-type *S. epidermidis* ATCC 12228 *and S. epidermidis* ATCC 12228 harboring an E105Q mutation in σ^A^ (see ‘Isolation of ureidothiophene resistant *S. epidermidis*’ below). RNAPs were purified as described above for *S. aureus*, apart from the polyethylenimine and ammonium sulphate precipitation steps were omitted, and holoenzymes were collected during ion exchange chromatography.

*Escherichia coli* σ^70^, *S. aureus* σ^A^ and *T. aquaticus* σ^A^ were expressed in T7 express cells (New England Biolabs) transformed with pET28 expression vector encoding N-terminally 6xHis-tagged σ subunits. Expression was induced by addition of 0.4 mM IPTG to exponentially growing cells and cells incubated on an orbital shaker (150 rpm) at room temperature overnight. Cells were then harvested by centrifugation and resuspended in grinding buffer. Cells were lysed by sonication and debris cleared by centrifugation. σ subunits were then purified on HisTrap HP column (GE Healthcare). Urd resistant mutations were introduced in the expression vector by site-directed mutagenesis using QuikChange II kit (Stratagene) and mutant proteins purified as above.

### *In vitro* transcription on promoter DNA

Transcription from promoter DNA fragments was performed essentially as described (20). Briefly, reactions were performed in 10 μl of transcription buffer TB (20 mM Tris–HCl pH 7.9, 40 mM KCl, 10 mM MgCl_2_). 1 pmol of *E. coli* RNAP core with 3 pmol of σ^70^ or 1 pmol of *T. aquaticus* RNAP core with 3 pmol of *T. aquaticus* σ^A^ or 1 pmol of *S. aureus* RNAP core with 3 pmol of *S. aureus* σ^A^ or 1 pmol of *M. smegmatis* or *S. epidermidis* RNAP holoenzymes were incubated in TB with 1 μl of DMSO containing or not containing Urd at 37°C (or 60°C in case of *T. aquaticus* RNAP) for 5 min. Transcription was initiated by the addition of 2 μl mixture of nucleotides and promoter DNA in TB, containing (final concentrations): 10 nM promoter DNA, 25 μM CpA (for T7A1 and GalP1 promoters) or 100 μM ApA (for lacUV promoter), 0.2 μl α-[^32^P]UTP (10mCi/ml) (Hartmann Analytic), 10 μM UTP with (run off transcription) or without (abortive transcription) 100 μM ATP, CTP and GTP. Reactions were stopped after 10 min incubation at 37°C (or 60°C in case of *T. aquaticus* RNAP) for run off transcription or 5 min for abortive transcription by the addition of equal volume of formamide-containing loading buffer. Products were resolved in denaturing polyacrylamide gels, revealed by PhosphorImaging (GE Healthcare), and analyzed using ImageQuant software (GE Healthcare)

### *In vitro* transcription on M13ori hairpin

*In vitro* transcription on single-stranded M13ori minimal promoter was performed as described in (10). Briefly, reactions were performed in 20 μl of TB. 3 pmol of RNAP core with 15 pmol of σ^70^ were incubated in TB with 2 μl of DMSO containing or not containing Urd, Fdx or Rip at 37°C for 5 min. 3 pmol of M13ori promoter (IDT) were then added to the reaction and incubated at 37°C for 10 min. Transcription was initiated by the addition of 1 mM ATP, CTP and UTP, 100 μM GTP and 0.2 μl α-[^32^P]GTP (10mCi/ml) (Hartmann Analytic). Reactions were stopped after 30-min incubation at 37°C by the addition of formamide-containing loading buffer. Products were analyzed as above.

### *In vitro* transcription in assembled elongation complexes

Elongation complexes were assembled as previously described (22). Sequences of oligonucleotides (IDT) used in assembly are illustrated in their corresponding figures. RNA was 5′ radiolabeled by T4 Polynucleotide Kinase and γ-[^32^P]-ATP prior to complex assembly, as described (22). Reactions were carried out in 15 μl (final volume) of TB. 0.5 pmol of 5′ labelled RNA and 1 pmol of template DNA were incubated in TB at 45°C for 5 min and then cooled slowly to 4°C. 5 pmol of core RNAP were added for 5 min at 37 °C. The complexes were then incubated with 10 pmol of non-template DNA bearing a 5′biotin tag for 5 min at 37°C. The complexes were then immobilized on 5 μl of streptavidin beads slurry, and washed first with TB containing high salt (1M KCl) and then TB. Reactions were then activated with 1 μM GTP or a combination of 1 μM GTP, CTP, UTP and ATP and incubated at 37°C for the times indicated in the respective figures. Reactions were stopped by the addition of formamide-containing loading buffer. Products were analyzed as above.

### KMnO_4_ and DNAse I footprinting

Reactions were performed in 20 μl final volume. Firstly, 5 pmol RNAP core and 10 pmols of σ^70^ were incubated in TB. For DNAse I footprinting, 2 μl of DMSO containing or not containing Urd was added to the reaction and incubated at 37°C for 5 min. For KMnO_4_ footprinting, 2 μl of 75% EtOH containing or not containing Urd was added to the reaction and incubated at 37°C for 5 min. Reactions were supplemented with 0.25 pmol of promoter DNA radiolabelled at the 5′ end of the non-template strand, as described (23), and incubated for a further 2 min at 37°C. Samples were then treated with 0.25 units of DNAse I (Roche) or 5 mM KMnO_4_ and incubated at 37°C for 30 s. For DNAse I footprinting, reactions were stopped with equal volume formamide-containing loading buffer. For KMnO4 footprinting, the reactions were stopped with an equal volume of 2-mercaptoethanol. The KMnO_4_ treated samples were then subjected to phenol–chloroform extraction and treated with piperidine. The samples were then further subjected to chloroform extraction, ethanol precipitated and dried before resuspension in formamide-containing loading buffer, as described (23). Products were resolved on denaturing polyacrylamide gels, and analyzed as above.

### Electrophoretic mobility shift assay (EMSA)

Reactions were performed in 20 μl final volume of EMSA Buffer (20 mM Tris–HCl pH 7.9, 40 mM KCl, 10 mM MgCl_2_, 5% glycerol). 2 pmol of RNAP core and 6 pmol of σ^70^ were incubated in EMSA buffer. 2 μl of DMSO containing or not containing Urd were added to the reaction and incubated at 37°C for 5 min. Reactions were supplemented with 0.2 pmol of promoter DNA radiolabelled as above and incubated for a further 5 min at 37°C. Next, heparin (50 μg/ml final) was or was not added, and samples incubated for a further 2 min at 37°C. Complexes were resolved in 4.5% non-denaturing polyacrylamide gel, which was dried and analyzed as above.

### Isolation of ureidothiophene resistant *S. epidermidis*

Firstly, Urd MIC of *S. epidermidis* ATCC12228 was assessed by serial dilution on a 24-well agar plate (dilutions from 100 μg/ml). Individual wells contained 1 ml of solid LB agar supplemented with 2.5% pluronic F68 (Thermofisher). Prior to agar setting, DMSO with or without Urd was added to the individual well to a final concertation of 5%. *S. epidermidis* ATCC12228 was streaked onto LB agar and grown at 37°C overnight. A single colony was picked and grown in liquid LB to 1 × 10^6^ CFU/ml. 10 μl of 10^6^ CFU/ml *S. epidermidis* inoculant were dotted onto each well and the plate incubated at 37°C overnight. MIC was deduced as the concentration in which no visible cell growth was observed (3.125 μg/ml). Secondly, *S. epidermidis* ATCC12228 was streaked onto standard LB agar and grown at 37°C overnight. A single colony was picked and grown in liquid LB until ∼1 × 10^9^ CFU/ml. 100 μl of 10^9^ CFU/ml *S. epidermidis* ATCC12228 was streaked onto an LB agar plate supplemented with 2.5% pluronic F68, containing 4× MIC of Urd (12.5 μg/ml). Urd resistant strain was confirmed by re-streaking on the same media, and sent for full Illumina genome sequencing (MicrobesNG). Genomes were assembled and SNPs identified by CLC Genomics Workbench software (Qiagen)

## RESULTS

### Urd inhibits a multitude of RNA polymerases *in vitro*

We assessed the effects of Urd on *in vitro* transcription by the wild-type *E. coli* RNAP, the most extensively characterized bacterial RNAP. When added to RNAP before DNA, Urd inhibited transcription on a linear DNA template, containing *lac*UV5 promoter (IC_50_ ∼15.1 ± 8.1μg/ml) (Figure 1B). A decrease in full length transcript synthesis coincided with a corresponding decrease in the synthesis of short abortive products. Indeed, Urd inhibits synthesis of both the tri- and tetra- nucleotide abortive products ApApU and ApApUpU in an abortive transcription assay (IC_50_ ∼18.5 ± 1.9 μg/ml) (Figure 1C). We further analyzed inhibition of abortive transcription on two more –10/–35 promoters, T7A1 and T7A2 with different promoter sequences, and the extended –10 *gal*P1 promoter which uses a TG motif upstream of –10 element instead of –35 element. Urd inhibited transcription on all of the tested promoters with similar IC50, indicating that the inhibition is not dependent on the promoter sequence or the presence of the –35 promoter element (Figure 1D, E). We assessed the ability of Urd to inhibit transcription by different bacterial RNAPs (Figure 1F). In this experiment we used T7A1 promoter, recognized by most of bacterial RNAPs. Consistently with previous observations (18), *S. aureus* RNAP was highly susceptible to Urd with an IC_50_ ∼ 0.79 (± 0.27) μg/ml. In contrast, *T. thermophilus* and *M. smegmatis* RNAPs were much less sensitive to Urd (Figure 1F). These observations suggest that Urd directly targets RNAP.

### Urd is an inhibitor of transcription initiation

**Figure 2.**
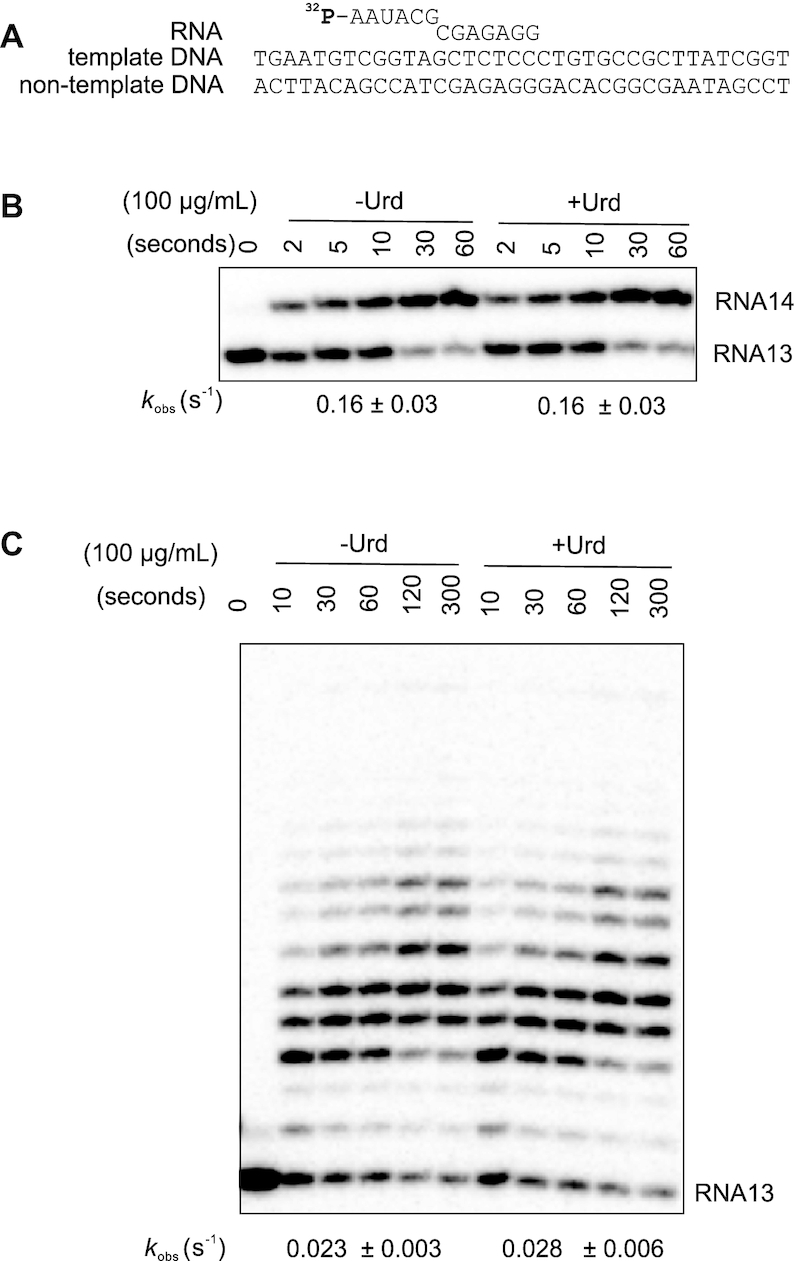
Ureidothiophene does not inhibit transcription elongation. (**A**) Scheme of the elongation complex scaffold used. RNA is 5′-[^32^P]-labelled. (**B** and **C**) Transcription elongation by one (GTP) or all four NTPs in the absence and presence of 100 μg/ml Urd. Rate constants are shown below the gels (numbers that follow the ± sign are standard errors).

Concurrent inhibition of both abortive and run-off transcription suggests Urd may inhibit nucleotide binding or catalysis. Therefore we analyzed the ability of Urd to inhibit single and multiple nucleotide addition by elongation complexes formed by *E. coli* RNAP core enzyme. Elongation complexes were assembled with fully complementary template and non-template strands and 5′-radiolabeled RNA (Figure 2A). As seen from Figures 2B, C, even high concentration (100 μg/ml) of Urd had no effect on RNA extension indicating that the inhibitor does not effect NTP binding or catalysis. This suggests that Urd targets early stages of transcription cycle.

### Urd prevents RNAP interaction with promoter DNA downstream of -35 element

**Figure 3.**
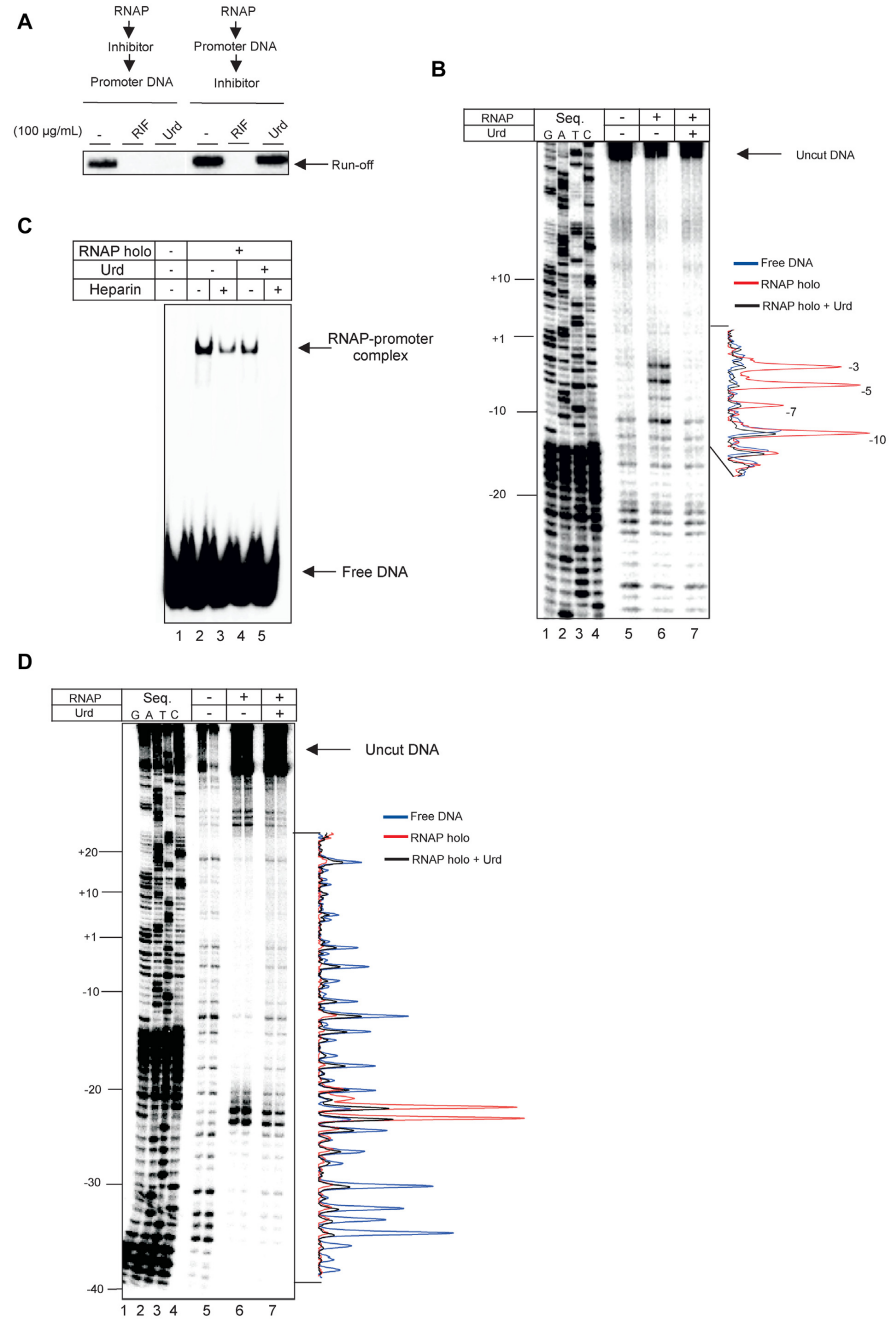
Urd prevents recognition of the -10 promoter element. (**A**) Effect of order of addition of Urd, prior to or following the formation of RPo, on inhibition of transcription by *E. coli* RNAP holoenzyme. (**B**) KMnO_4_ probing of RNAP-*lac*UV5 promoter complexes assembled in the absence and presence of 100 μg/ml Urd. Non-template strand is 5′-[^32^P]-labelled. Quantified profiles of the gel lanes are shown to right of the gel. Position of thymines susceptible to modification by KMnO_4_ in RPo are indicated. (**C**) Electrophoretic mobility shift assay (EMSA) of RNAP-*lac*UV5 promoter complexes assembled in the absence and presence of 100μg/ml Urd. Complexes were further challenged with heparin as indicated. (**D**) DNAase I probing of RNAP-*lac*UV5 promoter complexes assembled in the absence and presence of 100 μg/ml Urd. Non-template strand is 5′-[^32^P]-labelled. Quantified profiles of the gel lanes are shown to right of the gel.

When added after RPo formation, Urd does not have any effect on transcription (Figure 3A), indicating that it inhibits some step of the RPo formation. We analyzed if Urd targets formation of the RPo by KMnO_4_ footprinting, which probes the unpaired thymidine residues in the melted region of the RPo. Linear DNA fragment carrying *lac*UV5 promoter was radiolabelled at the 5′ end of the non-template strand. As can be seen from Figure 3B, Urd (100 μg/ml) added before mixing RNAP and promoter DNA completely inhibited formation of RPo; thymidines in positions –10, –7, –5 and –3 that are melted in RPo, remained double-stranded in the presence of Urd.

Urd may altogether block interactions of RNAP with DNA or prevent crucial interactions of RNAP with promoter DNA that are required for either RPc formation, or melting of promoter DNA. In order to distinguish between these possibilities, we first analyzed RNAP-promoter complex formation by electrophoretic mobility shift assay (EMSA). *E. coli* RNAP holoenzyme was treated with Urd (100 μg/ml) and then incubated with radiolabeled *lac*UV5 promoter DNA. As can be seen from Figure 3C, Urd did not abolish interaction of RNAP with promoter DNA. However, a challenge with polyanion heparin leads to complete destruction of the complexes formed in the presence of Urd, as compared to partial destruction without inhibitor (Figure 3C, compare lanes 3 and 5). Heparin is known to have much smaller effect on the formed RPo than on any preceding intermediates (24,25) We therefore conclude that Urd blocks some stage of isomerization into RPo, but does not abolish recognition of promoter DNA by RNAP.

To understand the nature of Urd/RNAP/promoter complexes, we performed DNase I footprinting of promoter complexes in the absence or presence of Urd (100 μg/ml) added before mixing RNAP and the promoter DNA (*lac*UV5 with 5′-radiolabelled non-template strand). The results indicate that Urd does not cause a significant changes in protection in the upstream promoter regions from positions –39 to –25 (Figure 3D), suggesting that σR4.2 remains engaged with the –35 promoter element in the presence of Urd. However, large difference in protection pattern is observed downstream of the –35 promoter region. Hypersensitive sites at positions –23 and –24 on the non-template strand, that apparently arise from distortion of the 18 base pair spacer region between the –10 and –35 promoter elements (26), have diminished sensitivity to DNAse I digestion in the presence of Urd. Urd causes a strong deprotection of nearly all bases downstream of position –20 up to +18. Notably, –11 adenosine residue, essential for recognition of –10 element (3,4,27), is deprotected in the presence of Urd indicating the –10 element is unable to form stable contacts with σR2. We conclude that Urd does not inhibit binding of the –35 promoter element, but somehow affects binding of –10.

### Urd may not target the switch region 2 of β′

**Figure 4.**
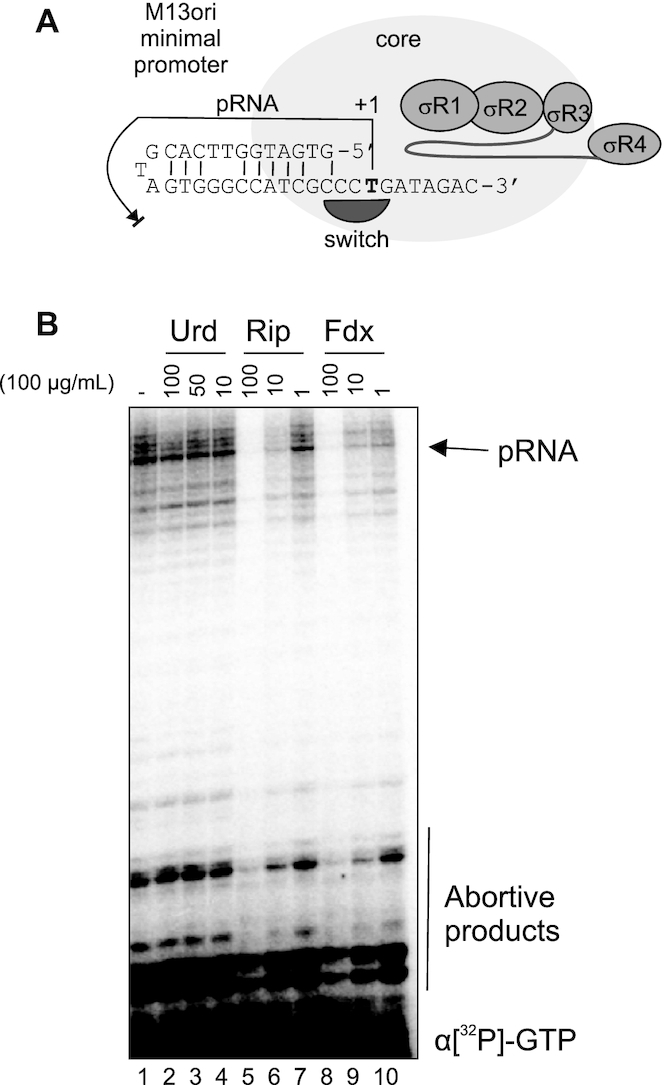
Urd does not affect downstream double-stranded DNA binding or single stranded DNA template loading into active center. (**A**) Structure of the single-stranded M13 minimal promoter recognized by downstream-DNA-binding channel of RNAP, and which binding does not depend on -10/-35 elements or on σ^70^ subunit. 18nt RNA product (pRNA) synthesized on M13ori promoter is shown with arrow. (**B**) Transcription on M13ori promoter by *E. coli* RNAP holoenzyme in the increasing concentrations of Urd, or β′ switch-2 targeting inhibitors Rip and Fdx.

Previous structural docking studies suggested that Urd may bind at the β′ switch-2 (17). To analyze if Urd may occlude the access of single stranded template DNA into the active site and/or preventing binding of duplex DNA into downstream DNA-binding channel, as do β′ switch-2 binders Fdx and Rip (see Introduction), we used single-stranded promoter of the origin of replication of M13 bacteriophage (M13ori; Figure 4A) (10,28,29). Formation of RPo on this unique promoter does not require usual promoter elements or σ subunit. M13ori forms a hairpin which is specifically recognized by the downstream DNA-binding channel of RNAP (Figure 4A). It lacks non-template DNA upstream of +2 position, and template DNA upstream of –7 position relative to transcription start site. Therefore, M13ori allows us to separate recognition of the promoter elements from the binding of DNA to downstream DNA-binding channel and loading of the single-stranded DNA into the active cleft. We compared transcription on M13ori that leads to the formation of 18nt primer RNA (pRNA; arrow in Figure 4A), in the presence of the inhibitors Urd, Fdx and Rip. As expected, Fdx and Rip strongly inhibit transcription on M13ori (Figure 4B) (12,14). Note that Fdx and Rip bind to different parts of β′ switch-2. However, Urd has no effect on formation of pRNA. Results indicate that, unlike Fdx and Rip (12,14), Urd does not occlude the binding of single stranded template DNA into the active site, does not prevent binding of downstream DNA duplex to the downstream DNA binding channel and does not prevent the conformational change of RNAP clamp, which is essential for downstream DNA binding in the RNAP main channel. This also suggests that Urd binding site is likely to be away from the β′ switch-2, in contrast to the earlier hypothesis (17). Taken together with DNase I footprinting results, these data suggest that Urd inhibits formation of a correct RPc that can isomerize into RPo by blocking interactions with the –10 promoter element.

### Urd may target σR1.2

**Figure 5.**
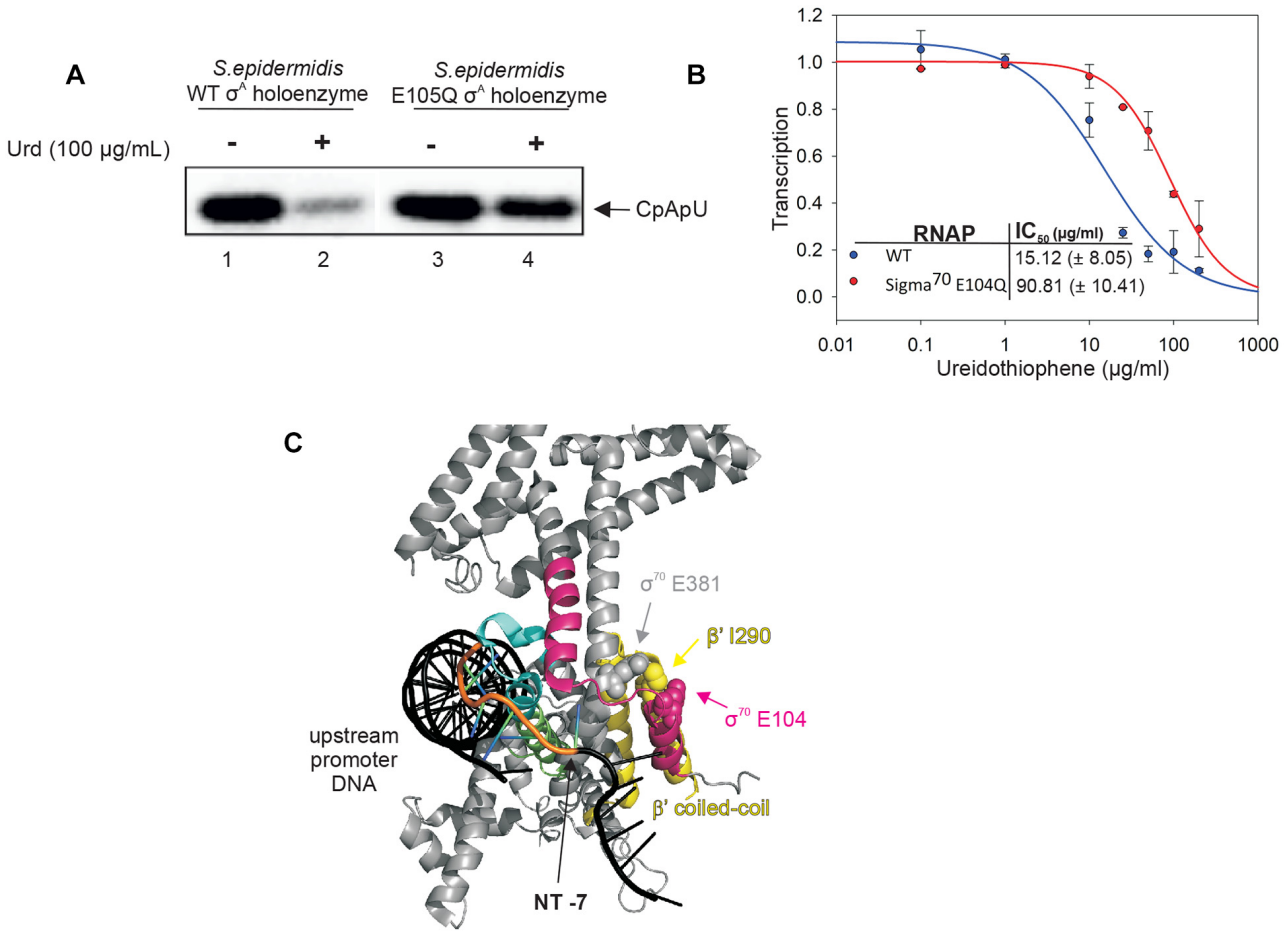
Urd inhibits RNAP by targeting σR1.2. (**A**) Abortive synthesis by wild-type and σ^A^E105Q *S. epidermidis* holoenzymes on T7A1 promoter in the absence and presence of 100 μg/ml Urd. (**B**) Inhibition by Urd of transcription by wild-type and σ^70^E104Q *E. coli* holoenzymes. Error bars are ± SD. (**C**) Recognition of the promoter -10 element within *E. coli* holoenzyme (PDB 6CA0) (32). σ^70^ is grey, and the RNAP β′ coiled-coil domain is yellow. σR1.2 (residues 96–126) is pink, σR2.3 (residues 416–434) is cyan, and 2.4 (residues 435–452) is green (3). Residues within the proposed σR1.2 allosteric switch are indicated in sphere model.

To delineate the putative binding site of Urd, we isolated an *S. epidermidis* (which was the most susceptible to Urd from available strains) spontaneous Urd resistant mutant conferring ≥4× resistance to the MIC of Urd (3.125 μg/ml). The mutant strain was resistant to Urd at >100 μg/ml. Genome sequencing revealed a single nucleotide substitution in the *rpoD* gene encoding the primary sigma factor σ^A^, Substitution resulted in E105Q mutation in σR1.2 of σ^A^ (Figure 5A). To confirm this mutation is responsible for the resistance phenotype, we purified RNAP holoenzyme from wild-type *S. epidermidis* and mutant *S. epidermidis* and analyzed sensitivity to Urd on the T7A1 promoter. Indeed, unlike the wild-type *S. epidermidis* RNAP, mutant RNAP holo bearing the E105Q mutation in σ^A^ subunit is resistant to inhibition by Urd (100 μg/ml) (Figure 5A). This result confirms that RNAP is a cellular target of Urd and also indicates the E105Q mutation underlies the observed resistance phenotype. To further corroborate this finding, we introduced the corresponding mutation into the *E. coli* σ^70^ subunit (E104Q in *E. coli* numbering used throughout below) and assessed the effect of the mutation on *E. coli* holoenzyme sensitivity to Urd. Mutant holoenzyme was ∼6-fold more resistant to Urd than the wild-type RNAP (Figure 5B). As σR1.2 is implicated in allosteric regulation of –10 element recognition, this result is consistent with the above conclusions on Urd mode of action.

## DISCUSSION

The principle finding of this study is a new mode of inhibition of bacterial transcription. We show that Urd inhibits interaction of RNAP with –10 promoter element and other parts of promoter DNA downstream of –35 element during RPc formation. We also show that the action of Urd is manifested through essential regulatory region of σ subunit, σR1.2.

DNA footprinting data suggests that, in the presence of Urd, σ cannot stably engage with –10 element and downstream part of promoter DNA, i.e. form a correct closed promoter complex. Urd, thus, acts at earlier stages of transcription initiation than do Rip, Cor and Myx, which target the β′ switch-2, and inhibit transformation of closed promoter complex into RPo (13,14). Urd may act in a similar way as Fdx, that, by binding to β′ switch-2, blocks conformational flexibility of RNAP that is required for engagement with –10 element (11). Although we cannot exclude the possibility that Urd also binds at the β′ switch-2, it must do so in a way distinct from the known inhibitors targeting β′ switch-2, including Fdx. This is evidenced from the differences in inhibition of initiation on M13 promoter that lacks non-template strand upstream of +2 position and template strand upstream of –7 position, which makes its binding by RNAP independent of –10 element (10,28,29). We show that overcoming the necessity of -10 element recognition on M13ori promoter confers resistance to Urd, but not to Fdx or Rip.

The dependence of Urd inhibition on σR1.2 integrity suggests the compound mediates inhibition of transcription by interacting with this particular σ factor sub-region or disrupt conformational change governed by this σ region, which is required for interaction of holoenzyme with downstream promoter DNA. σR1.2 has previously been shown to play a role in formation of stable open promoter complexes, in particular it is implicated in allosteric control of –10 promoter element recognition by σR2 (5–7). The amino acid E104 of σR1.2, mutation of which confers resistance of RNAP to Urd, was implicated in the formation of open promoter complexes (7). Neighboring Y101 of σR1.2 was shown to play a particularly important role in σR1.2 regulation of –10 element recognition (6,30). In the structures of *E. coli* RPo and holoenzyme, σR1.2 E104 interacts with residue I290 of the β′ coiled-coil domain, while the latter interacts with residue E381 of σR2. This may create a mechanical linkage between σR1.2 and σR2 (Figure 5C), and thus influence –10 promoter element binding by σR2. The preclusion of interaction of holoenzyme with the –10 promoter element by Urd suggests the inhibitor may target this ‘allosteric switch’ of σR1.2 that controls σR2. We cannot exclude that E104 is not the direct target of Urd, and that E104Q mutation makes local structural alterations that can be tolerated during transcription initiation, but preclude binding of Urd in the vicinity. It is also possible that σR1.2 is the integral part of structural rearrangement of RNAP in the process of engagement with -10 element, and the mutation in σR1.2, thus, may confer resistance to the Urd that binds elsewhere. Mutation in σR1.2 has been shown to affect sensitivity to Fdx, though with an opposite effect – mutation E116G increased sensitivity to Fdx (31). Further structural studies are required for unbiased elucidation of Urd binding site. Testing Urd with holoenzymes with different sigma subunits may also shed further light on the mechanisms of promoter complexes formation at different promoters and possibly open the way to new molecules targeting specifically virulence or other bacterial life choices.

## Supplementary Material

gkaa591_Supplemental_File
