# Supplementary figures and images for: Ureidothiophene inhibits interaction of bacterial RNA polymerase with –10 promotor element
Source: Nucleic Acids Res. 2020 Jul 11;48(14):7914–23. doi: 10.1093/nar/gkaa591 (PMC7430646; doi:10.1093/nar/gkaa591)

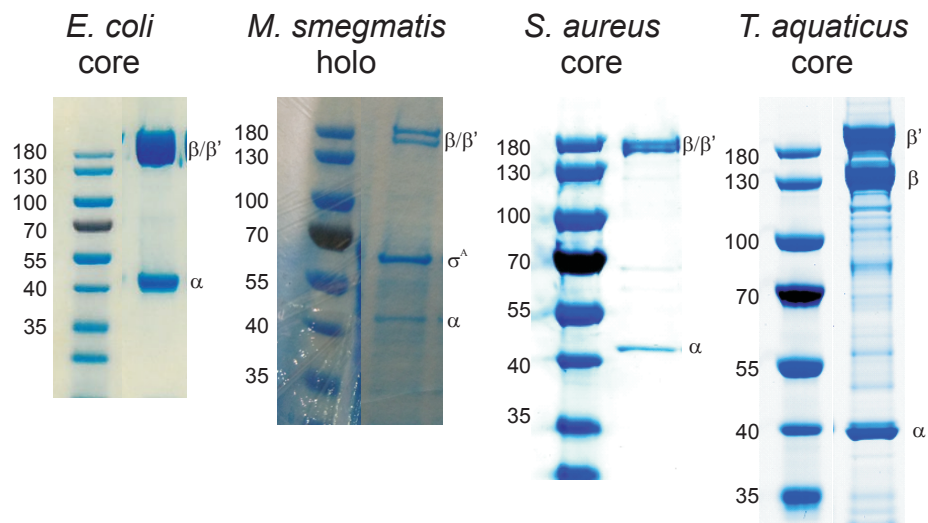

Figure S1. SDS-gels of the purified RNAP core and holoenzymes. Left lane in each gel is a size marker.

Supplement: gkaa591_Supplemental_File [file gkaa591_supplemental_file.pdf]
